# Loss of cyclin-dependent kinase inhibitor genes and chromosome 9 karyotypic abnormalities in human bladder cancer cell lines.

**DOI:** 10.1038/bjc.1995.488

**Published:** 1995-11

**Authors:** J. Southgate, J. Proffitt, P. Roberts, B. Smith, P. Selby

**Affiliations:** ICRF Cancer Medicine Research Unit, St. James's University Hospital, Leeds, UK.

## Abstract

**Images:**


© 1995 Stockton Press All rights reserved 0007-0920/95 $12.00

### Loss of cyclin-dependent kinase inhibitor genes and chromosome 9 karyotypic abnormalities in human bladder cancer cell lines

J Southgate[1], J Proffitt[1]*, P Roberts[2], B Smith[1] and P Selby[1]

[1]*ICRF Cancer Medicine Research Unit and* [2]*Cytogenetics Unit, St. James's University Hospital, Leeds LS9 7TF, UK.*

**Summary** Loss of cell cycle control through the structural or functional aberration of checkpoint genes and their products is a potentially important process in carcinogenesis. In this study, a panel of well-characterised established human bladder cancer cell lines was screened by the polymerase chain reaction for homozygous loss of the cyclin-dependent kinase inhibitor genes *p15*, *p16* and *p27*. The results demonstrate that, whereas there was no genetic loss of *p27*, homozygous deletion of both *p15* and *p16* genes occurred in seven of 13 (54%) independent bladder cell lines tested. Differential loss of either the *p15* or *p16* gene was not seen. The *p15* and *p16* genes are known to be juxtaposed on chromosome 9p21 at the locus of a putative tumour-suppressor gene involved in the initiation of bladder cancer. Cytogenetic analysis of the cell lines revealed karyotypes ranging from near diploid to near pentaploid with complex rearrangements of some chromosomes and a high prevalence of chromosome 9p rearrangements, although all cell lines contained at least one cytogenetically normal 9p21 region. These observations support a role for *p15/p16* gene inactivation in bladder carcinogenesis and/or the promotion of cell growth *in vitro* and lend support to the hypothesis that homozygous deletion centred on 9p21 is a mechanism by which both *p15* and *p16* genes are co-inactivated.

**Keywords**: cyclin-dependent kinase inhibitors; *p15*; *p16*; *p27*; bladder cancer; cell cycle; cytogenetics

Transitional cell carcinoma (TCC) is the most common cancer of the urinary bladder and orginates from the normal stratified transitional epithelium, the urothelium. The natural history of TCC is unclear as it is currently not possible to distinguish the 3% subgroup of patients with superficial non-invasive papillary disease who will progress to muscle-invasive malignant disease from those patients in whom the disease is less aggressive. It is thought that the homozygous loss of a tumour-suppressor gene may be the initiating factor in bladder cancer and interest has centred on chromosome 9, which shows a high rate of allelic loss that is both stage and grade independent (Cairns *et al.*, 1993; Miyao *et al.*, 1993; Ruppert *et al.*, 1993; Stadler *et al.*, 1994). Deletion mapping studies of primary bladder tumours indicate the presence of two independent tumour-suppressor loci on chromosome 9, located proximally on 9p and 9q (Ruppert *et al.*, 1993; Keen and Knowles, 1994). In two independent studies, the 9p locus has been mapped to the 9p21–22 region (Cairns *et al.*, 1994*a*; Stadler *et al.*, 1994).

The 9p21–22 region is also implicated in tumour development of a wide spectrum of other human tumours, including familial and sporadic melanoma (Cannon-Albright *et al.*, 1992; Fountain *et al.*, 1992; Hussussian *et al.*, 1994; Kamb *et al.*, 1994*a*). The location of the *p16 cyclin-dependent kinase 4 inhibitor* (*Cdk4I*) gene at 9p21 has led to the hypothesis that inhibitors of $G_1/S$ cell cycle progression may show tumour-suppressor function and hence have oncogenic activity if homozygously lost through deletion and/or mutation (reviewed by Hartwell and Kastan, 1994; Marx, 1994*a*). In addition to p16 (Serrano *et al.*, 1993), three other Cdk inhibitors have been identified: p15 (Hannon and Beach, 1994), p21 (El-Diery *et al.*, 1993; Harper *et al.*, 1993; Xiong *et al.*, 1993*a*) and p27 (Polyak *et al.*, 1994*a*; Toyoshima and Hunter, 1994) and these too have the potential to act as tumour-suppressors or to confer growth advantage *in vitro*. Transcription of *p21* is stimulated by p53 (El-Deiry *et al.*, 1993) and by cell senescence (Noda *et al.*, 1994) and expression of p21 protein is reduced in cells with null p53 (Xiong *et al.*, 1993*b*). The p15 and p27 inhibitors are each implicated in TGF-$\beta$-mediated $G_1$ phase arrest (Hannon and Beach, 1994; Polyak *et al.*, 1994*b*). The potential role of the *p27* gene in cancer is, as yet, undetermined.

A number of cell lines have been established from bladder cancers, and these have been used extensively as *in vitro* models of neoplastic urothelial cell behaviour (reviewed Masters *et al.*, 1986). Bladder cancer cell lines typically show complex karyotypes that reflect the genesis and evolution of the cancer and also the selection pressures imposed during adaptation to long-term survival *in vitro*. We have used the polymerase chain reaction (PCR) to screen a panel of well-characterised bladder cancer cell lines for homozygous loss of *p15*, *p16* and *p27* gene sequences.

### Materials and methods

### *Cell lines*

In total, 17 established human bladder cell carcinoma cell lines were used: RT4, RT112, HT1376, HT1197, COLO232, KK47, VM-CUB-1, VM-CUB-3, 253J, EJ, HU456, HU961T, MGH-U2, T24, TCCSUP, HCV29 and SCaBER. These cell lines have been reviewed and referenced by Masters *et al.* (1986). The cell lines were maintained in a 1:1 mixture of RPMI-1640 and Dulbecco's modified Eagle medium (DMEM) with 5% fetal bovine serum (FBS), as described previously (Trejdosiewicz *et al.*, 1985).

### *Cytogenetic analysis*

Cultures of bladder cancer cell lines in exponential growth were exposed to 0.2 $\mu$l ml$^{-1}$ colcemid (Sigma) in growth medium for 17 h. Cells were removed from the substrate with a solution of 0.25% (w/v) trypsin and 0.02% (w/v) EDTA in phosphate-buffered saline (PBS) for 5 min and incubated for 15 min in 0.075 M potassium chloride before washing three times in Carnoy's fixative. Slides were analysed by GTL banding and 3–6 metaphases were examined for each cell line.

### *DNA extraction*

DNA extractions were performed using standard procedures involving digestion with proteinase K in the presence of sodium dodecyl sulphate (SDS) followed by phenol/chloroform extractions and ethanol precipitation. The integrity of DNA for each cell line was confirmed by PCR for an

Correspondence: J Southgate
*Present address: Department of Biochemistry and Molecular Biology, University of Leeds, Leeds LS2 9JT, UK
Received 1 May 1995; revised 19 June 1995; accepted 23 June 1995

unrelated gene (below) and the same DNA preparation was used in all subsequent analyses. The passage number of the cell line stocks used is shown in Table I.

### *Arbitrary primed PCR (AP-PCR)*

AP-PCR was used to obtain genomic 'fingerprints' of the cell lines and was performed on 50 ng DNA in a 25 µl reaction volume containing 10 mM Tris pH 8.3, 50 mM potassium chloride, 0.125 mM each dNTP, 5 mM magnesium chloride, 1.6 µM oligonucleotide, 1 µCi [$^{32}$P]α-dATP and 1 U AmpliTaq (Perkin Elmer). After incubation at 94°C for 4 min, five cycles of PCR were performed at 94°C for 1 min, 35°C for 1 min and 72°C for 1 min, followed by 30 cycles at 95°C for 1 min, 50°C for 1 min and 72°C for 1 min with a final extension at 72°C for 10 min. Samples were run on 5% polyacrylamide sequencing gels. Random oligonucleotide sequences of 20–28 base length were used to generate DNA banding fingerprints. The results described here refer specifically to oligonucleotide BO11 (Table II).

### *PCR*

PCR was performed on 100 ng each of genomic DNA extracted from the human bladder cell lines and from human placental DNA (Sigma) included as positive control. Primer sequences are shown in Table II.

For amplification of *p15*, primers p15(2)F and p15(2)R were used to amplify a 430 bp product from exon 2 of the *p15* gene, as described by Jen *et al.* (1994). The reaction mix consisted of 67 mM Tris pH 8.8, 16.6 mM ammonium sulphate, 6.7 mM magnesium chloride, 10 mM β-mercaptoethanol, 6% (v/v) dimethylsulphoxide (DMSO), 1.25 mM dNTPs, 1 µM of each oligonucleotide primer and 5 U of AmpliTaq. The PCR cycling conditions were denaturation for 2 min at 95°C followed by 35 cycles of denaturation at 95°C for 30 s, reannealing at 66°C for 1 min and extension at 70°C for 1 min, with a final extension at 70°C for 5 min.

For all other PCR reactions, each 50 µl reaction mix contained 10 mM Tris pH 8.3, 50 mM potassium chloride, 0.2 mM dNTPs, 2 mM magnesium chloride, 1 µM of each oligonucleotide primer (except for *p16*, where 0.15 µM of each oligonucleotide was used) and 1 U AmpliTaq. *p16* primers were chosen that span an intron–exon boundary of the human Cdk4I (*p16*) gene and amplify a 167 bp product by PCR (Nobori *et al.*, 1994). PCR cycling conditions were denaturation at 95°C for 1 min, reannealing at 62°C for 30 s and extension at 72°C for 30 s for a total of 40 cycles, with a final extension at 72°C for 10 min. In all cases where a negative results was obtained, the reaction was repeated.

PCR primers, p27AF and p27AR, were selected for the human *p27* gene from cDNA sequence available from the Genbank database (accession number U10906). The PCR cycling profile for *p27* was denaturation at 95°C for 1 min, reannealing at 60°C for 30 s and extension at 72°C for 30 s for a total of 40 cycles, with a final extension at 72°C for 10 min.

The integrity of DNA preparations was checked with primers HMIP1 and HMIP2 which amplify a 682 bp product from the 5' flanking region of the human *LD78α* gene located on chromosome 17q (Hirashima *et al.*, 1992). The PCR cycling profile for the *LD78α* primers was denaturation at 95°C for 1 min, reannealing at 60°C for 1 min and extension at 72°C for 1 min for a total of 30 cycles, with a final extension at 72°C for 10 min.

PCR products from individual experiments were pooled and run on 2% agarose gels and visualised with UV light after staining with ethidium bromide. The 186 bp PCR product obtained with the *p27* primers was confirmed as the correct sequence by blotting the gel onto Hybond N (Amersham International) and hybridising with oligonucleotide p27BF derived from the internal sequence.

**Table I** PCR amplification of DNA from bladder cancer cell lines

| *Cell line* | *PCR product* | | | |
|---|---|---|---|---|
| *passage no.*[a] | p15 | p16 | p27 | LD78α |
| COLO232 (5) | – | – | + | + |
| EJ 72[b] | + | + | + | + |
| HCV29 31 | – | – | + | + |
| HT1197 63 | + | + | + | + |
| HT1376 70 | + | + | + | + |
| KK47 24 | + | + | + | + |
| RT4 61 | – | – | + | + |
| RT112 77 | – | – | + | + |
| SCaBER (4) | + | + | + | + |
| VM-CUB-1 (3) | – | – | + | + |
| VM-CUB-3 27 | – | – | + | + |
| TCCSUP 70 | + | + | + | + |
| 253J 94 | – | – | + | + |

[a]Where absolute passage number is unknown, number of passages since receipt of cell line is shown in parentheses. [b]Same result found for MGH-U2, HU961T, T24 and HU456.

### Results

### *AP-PCR*

Comparison of genomic 'fingerprints' generated by arbitrary primed PCR revealed a single band loss from the EJ, MGH-U2, HU961T, T24 and HU456 cells which was retained in the other cell lines (Figure 1). These results are consistent with earlier reports based respectively on isoenzyme analysis and minisatellite probes which suggested cross-contamination of these particular cell lines (O'Toole *et al.*, 1983; Masters *et al.*, 1988).

### *Cytogenetic analysis*

Although the karyotypes of the cell lines have been previously reported, it was felt important to obtain specific karyotypes for the sublines used in this study in order to circumvent problems of genotypic drift. Cytogenetic analysis of the cell lines revealed karyotypes ranging from near di-

**Table II** Oligonucleotide sequences

| *Gene* | *Primer* | *Position* | *Sequence* |
|---|---|---|---|
| *p15* | p15(2)F | Forward | 5'-CCTTAAATGGCTCCACCTGC-3' |
| | p15(2)R | Reverse | 5'-CGTTGGCAGCCTTCATCG-3' |
| *p16* | p16f | Forward | 5'-GGAAATTGGAAACTGGAAGC-3' |
| | p16r | Reverse | 5'-CTGCCCATCATCATGACCTG-3' |
| *p27* | p27AF | Forward | 5'-CAAACGTGCGAGTGTCTAAC-3' |
| | p27AR | Reverse | 5'-AATCGAAATTCCACTTGCGC-3' |
| | P27BF | Internal | 5'-CAGAGACATGGAAGAGGCGA-3' |
| *LD78α* | HMIP1 | Forward | 5'-CTAGGCACCTGACATATTGAC-3' |
| | HMIP2 | Reverse | 5'-TTCTGAGCAGGTGACGGAATG-3' |
| – | BO11 | – | 5'-GTCAGTTAAGCAGGAAGGGACTAAC-3' |

**Figure 1** AP-PCR analysis of DNA from human bladder tumour cell lines. AP-PCR was performed with primer BO11 and the products run on a 5% polyacrylamide sequencing gel. The arrow locates the position of the band that is lost in the cell lines thought to be derived from the same originating cell line. T24 (not shown on the gel) also showed loss of the same band. Lane 1, normal human placental DNA; lane 2, EJ; lane 3, RT112; lane 4, HU456; lane 5, HT1197; lane 6, HCV29; lane 7, HT1376; lane 8, SCaBER; lane 9, VM-CUB-3; lane 10, VM-CUB-1; lane 11, HU961; lane 12, KK47; lane 13, COLO232; lane 14, MGH-U2; lane 15, RT4; lane 16, normal human placental DNA.

ploid to near pentaploid with complex rearrangements of some chromosomes. Chromosome 9p was targeted as a region of special interest and the results are summarised in Table III and illustrated in Figure 2.

*Detection of cyclin-dependent kinase inhibitor gene sequences*

These results are summarised in Table I and illustrated in Figure 3. Using the specific *p16* primers, the expected 167 bp-sized single product was amplified by PCR from human placental DNA (included as a positive control) and with DNA from ten of the 17 bladder cell lines. Using the same optimised PCR conditions, no product could be amplified from any of the remaining seven cell lines (RT4, RT112, VM-CUB-1, VM-CUB-3, COLO232, 253J and HCV29). The ten *p16*-positive cell lines included all five of the cell lines suspected of cross-contamination. When only independent cell lines were considered, 6/13 were positive for *p16*.

**Figure 2** Partial karyotypes of four human bladder tumour cell lines to illustrate normal and rearranged copies of chromosome 9.

The specific *p15* primers amplified the expected single 430 bp product from the control human placental DNA and from the same ten bladder cell lines which were positive for *p16* (above), including all five cell lines suspected of cross-contamination. All seven *p16*-negative cell lines failed to amplify any product with the *p15*-specific primers.

**Table III** Chromosome 9 abnormalities in bladder cancer cell lines

| *Cell line* | *Chromosome no. and ploidy* | *Expected no. of 9's* | *Observed no. of 9's* | *No. of normal 9's* | *Rearrangement affecting 9p* | *Other 9 rearrangements not affecting 9p* | *9p status* |
|---|---|---|---|---|---|---|---|
| HCV29 | 47 near diploid | 2 | 1 | 1 | – | – | Monosomy |
| COLO232 | 55 hyperdiploid | 2 | 2 | 1 | dic (7;9)(p13;p21-22) | – | Loss of 9p21-22→9pter |
| SCaBER[a] | 106-110 near pentaploid | 5 | 6 | 4 | 2 copies of der (9;14)(q10;q10) | – | Loss of all 9p in 2/6 9's |
| VM-CUB-3 | 46 diploid | 2 | 2 | 2 | – | – | Unaffected[b] |
| TCCSUP[a] | 71-73 near triploid | 3 | 3 | 3 | – | – | Unaffected[b] |
| KK47[a] | 58 near triploid | 3 | 2 | 1 | 9p21-22 rearrangement | – | 9p21-22 terminal deletion + other possibles |
| EJ[a] | 91 near tetraploid | 4 | 4 | 2 | 2 × 9's with del (9)(p21-22:) | – | 9p21-22 terminal deletion in 2/4 9's |
| RT4 | 79-82 near tetraploid | 4 | 3 | 2 | marker with most of 9q but no 9p | – | Loss of 9p |
| HT1197[a] | 74-79 near triploid | 3 | 4 | 1 | 2 × 9's with der(9;?)(q10;?) and marker chromosome with loss of all 9p | – | Loss of 9p in 3/4 9's |
| 253J | 59 near triploid | 3 | 3 | 3 | – | – | Unaffected[b] |
| HT1376[a] | 104-107 near pentaploid | 5 | 4-6 | 3-4 | i(9q) | Two copies 9q rearrangement | Loss of 9p in 1/5 9's |
| RT112 | 47 near diploidy | 2 | 2 | 1 | – | Rearrangement of 9q34 | Unaffected[b] |
| VM-CUB-1 | 75 near triploid | 3 | 3 | 2 | der (9;14)(q10;q10) | – | Loss of 9p in 1/3 9's |

[a]Indicates cell lines from which p16 was amplified by PCR. [b]No change in 9p.

**Figure 3** PCR analysis of human bladder tumour cell line DNA. (**a**) Agarose gel showing 430 bp PCR product obtained with primers specific for *p15*. (**b**) Products of PCR performed with primers specific for *p16* and *LD78α* gene sequences were pooled for each cell line and run on an agarose gel. The top band corresponds to the 682 bp *LD78α PCR* product. The lower band corresponds to the 167 bp *p16* PCR product. (**c**) Products of PCR performed with primers specific for *p27* and *LD78α* gene sequences were pooled for each cell line and run on an agarose gel. The top band corresponds to the 682 bp *LD78α* PCR product. The lower band corresponds to the 186 bp *p27* PCR product which is present in all cell lines. (**d**) Hybridisation of Southern blot of gel B with primer B27BF. Lane 1, control human placenta DNA; lane 2, COLO232; lane 3, EJ; lane 4, HCV29; lane 5, HT1197; lane 6, HT1376; lane 7, HU456 (equivalent to EJ cells – see text); lane 8, KK47; lane 9, RT4; lane 10, RT112; lane 11, SCaBER; lane 12, VM-CUB-1; lane 13, VM-CUB-3; lane 14, TCCSUP; lane 15, 253J.

Using the *p27* primers, a single PCR product of 186 bp was amplified from control DNA and from all of the 13 independent bladder cell lines. The integrity of all the genomic DNA preparations was confirmed by successful amplification of a 682 bp product from the 5′ flanking region of the human *LD78α* gene on chromosome 17q.

### Discussion

Recent advances in understanding how regulation of the cell cycle is achieved through the coordinated activity of cyclin-dependent kinases and checkpoint controls has revealed groups of genes with hitherto unforeseen oncogenic and tumour-suppressor potential (Hartwell and Kastan, 1994; Marx, 1994*a*). The *Cdk41* (*p16*) gene is one such candidate and resides within the critical deleted region 9p21–22 implicated in tumour development progression of a wide spectrum of human tumours. In particular, the *p16* gene is homozygously lost with high frequency in cell lines derived from a large range of human tumours, including bladder (Kamb *et al.*, 1994*b*; Nobori *et al.*, 1994). This has important implications for bladder cancer as the grade and stage independence of the chromosome 9p changes would suggest that, if *p16* is the putative tumour suppressor, deregulation of cell cycle control is an important initiating event in human bladder cancer. However, the apparently higher frequency of *p16* loss in bladder cell lines compared with the primary tumour counterparts (Cairns *et al.*, 1994*b*; Spruck *et al.*, 1994) has thrown the role of *p16* as a major multiple tumour-suppressor gene into some dispute (Bonetta, 1994; Marx, 1994*b*; Wainwright, 1994). An alternative hypothesis is that loss of *p16* confers a selective growth advantage during adaptation of tumour cells to culture and hence is important in the generation of bladder cancer cell lines (Cairns *et al.*, 1994*b*; Spruck *et al.*, 1994; Yeager *et al.*, 1995). A definitive answer to this debate is hindered by the problems of isolating tumour cell DNA uncontaminated by normal stromal cell DNA.

In this study, homozygous loss of the *p15* and *p16* genes was associated with 54% (7 13) bladder cancer cell lines. Kamb *et al.* (1994*b*), have previously reported homozygous *p16* gene deletions from 33% (5 15) bladder cancer cell lines but did not name the cell lines used. The potential problems of long term cross-contamination of cell lines underlines the importance of individual cell line pedigree in such studies. The cell lines MGHU2, HU961, T24, HU456 and EJ, although received originally (*c*. 1978) from different sources, were revealed to have identical DNA banding patterns on AP-PCR-generated fingerprints and furthermore, have previously been reported to derive from one originating cell line (O'Toole *et al.*, 1983; Masters *et al.*, 1988). Had these five cell lines been independently included in the *p16* gene analysis, the results would have suggested *p16* gene loss from only 41% (7 17) of lines.

Our findings agree well with Spruck *et al.* (1994), who also found *p16* loss in 54% bladder cancer cell lines. Of the 13 cell lines studied by Spruck *et al.* (1994) five were duplicated in our study (RT4, HT1376, TCCSUP, SCaBER, T24/EJ) and of these, the SCaBER line was revealed to contain a mutated *p16* sequence. When combined, the data from these two studies suggest that from a total of 21 cell lines, *p16* was homozygously lost from 62% (13) bladder cancer cell lines. It has recently been suggested that the high incidence of homozygous deletions around the 9p21 region may be a mechanism by which both *p16* and the neighbouring *p15* gene are efficiently and simultaneously inactivated (Jen *et al.*, 1994) and our results support this hypothesis.

Cytogenetic analysis has confirmed the frequent involvement of chromosome 9p in bladder cancer cell lines, reminiscent of the involvement of chromosome 11p rearrangements in Wilms' tumours (reviewed by Junien and Henry, 1994). Nevertheless, the seven *p16*-negative bladder cancer cell lines all contained at least one cytogenetically normal 9p21 region, demonstrating that loss of the *p16* gene was too small to be detected cytogenetically. This is consistent with the data of Nobori *et al.* (1994) who found that deletions at 9p21 in tumour cell lines were centred on the *p16* gene and were often small. In terms of the likely succession of genotypic changes, it seems probable that many of the chromosome 9p rearrangements preceded changes in ploidy, as three cell lines (SCaBER, EJ and HT1197) revealed more than one copy of the same derivative 9p chromosome and three *p16*-negative cell lines (VM-CUB-1, RT4 and 253J) contained more than one cytogenetically normal copy of chromosome 9. It is also worth noting that chromosome 9p rearrangements were detected in five of the six cell lines from which a *p16* PCR product was amplified: further studies will be needed to determine whether these cell lines actually transcribe and translate a functional *p16* gene product before this can be taken as *prima facie* evidence for alternative tumour-suppressor genes residing at 9p21–22 in bladder cancer (Bonetta, 1994; Spruck *et al.*, 1994).

The adaptation of tumours to growth *in vitro* places rigorous selection pressures on the individual tumour cells. Indeed, compared with normal bladder epithelium (Southgate *et al.*, 1994), the majority of primary bladder tumours do not readily adapt to *in vitro* growth (Niell and Soloway, 1983, and unpublished observations). Derangement of cell cycle control through loss of Cdk inhibitor function may provide an adaptive growth advantage *in vitro*. Expression of the p53-associated Cdk inhibitor protein, p21, has been shown to be reduced in cells with *p53* mutations (Xiong *et al.*, 1993). Thus, whereas the *p21* gene is not a direct target, loss of expression through *p53* mutation can result in a phenotypic loss of suppressor function. Spruck *et al.* (1994) showed that, of the six bladder cancer cell lines that were *p16* positive, only one cell line contained a wild-type *p53* gene. Homozygous loss of p53 is a relatively late event in the progression of bladder cancer (Sidransky *et al.*, 1991) and, as yet, it is unknown whether loss of *p16* or *p21* function could offer differential survival benefits *in vitro*. Our studies suggest that the *p27* gene is not commonly homozygously deleted in human bladder cancers. However, further studies will be needed to determine whether loss of p27 protein function is important either in bladder tumour progression and/or in the evolution of bladder tumour cell lines.

**Acknowledgements**
We are grateful to the Imperial Cancer Research Fund and the Yorkshire Cancer Research Campaign for financial support. We thank P Horsfield, S Morris and N Telford at the Cytogenetics Unit for expert assistance in cytogenetics.

### References

BONETTA L. (1994). Open questions on *p16*. *Nature*, **370**, 180.
CAIRNS P. SHAW ME AND KNOWLES MA. (1993). Initiation of bladder cancer may involve deletion of a tumour-suppressor gene on chromosome 9. *Oncogene*, **8**, 1083-1085.
CAIRNS P. TOKINO K. EBY Y AND SIDRANSKY D. (1994*a*). Homozygous deletions of 9p21 in primary human bladder tumors detected by comparative multiplex polymerase chain reaction. *Cancer Res.*, **54**, 1422-1424.
CAIRNS P. MAO L. MERLO A. LEE DJ. SCHWAB D. EBY Y. TOKINO K. VAN DER RIET P. BLAUGRUND JE AND SIDRANSKY D. (1994*b*). Rates of *p16* (MTS1) mutations in primary tumors with 9p loss. *Science*, **265**, 415-416.
CANNON-ALBRIGHT LA. GOLDGAR DE. MEYER LJ. LEWIS CM. ANDERSON DE. FOUNTAIN JW. HEGI ME. WISEMAN RW. PETTY EM. BALE AE. OLUNMILAYO OI. DIAZ MO. KWIATKOWSKI DJ. PIEPKORN MW. ZONE JJ AND SKOLNICK MH. (1992). Assignment of a locus for familial melanoma, MLM, to chromosome 9p13-p22. *Science*, **258**, 1148-1152.
EL-DEIRY WS. TOKINO T. VELCULESCU VE. LEVY DB. PARSONS R. TRENT JM. LIN D. MERCER WE. KINZLER KW AND VOGELSTEIN B. (1993). WAF1, a potential mediator of p53 suppression. *Cell*, **75**, 817-825.
FOUNTAIN JW. KARAYIORGOU M. ERNSTOFF MS. KIRKWOOD JM. VLOCK DR. TITUS-ERNSTOFF L. BOUCHARD B. VIJAYASARADHI S. HOUGHTON AN. LAHTI J. KIDD VJ. HOUSMAN DE AND DRACOPOLI NC. (1992). Homozygous deletions of human chromosome 9p21 in melanoma. *Proc. Natl Acad. Sci. USA*, **89**, 10557-10561.
HANNON GJ AND BEACH D. (1994). p15$^{ink4b}$ is a potential effector of TGF-$\beta$-induced cell cycle arrest. *Nature*, **371**, 257-261.
HARPER JW. ADAMI GR. WEI N. KEYOMARSI K AND ELLEDGE SJ. (1993). The *p21* Cdk-interacting protein Cip1 is a potent inhibitor of $G_1$ cyclin-dependent kinases. *Cell*, **75**, 805-816.
HARTWELL LH AND KASTAN MB. (1994). Cell cycle control and cancer. *Science*, **266**, 1821-1828.
HIRASHIMA M. ONO T. NAKAO M. NISHI H. KIMURA A. NOMIYAMA H. HAMADA F. YOSHIDA M AND SHIMADA K. (1992). Nucleotide sequence of the third cytokine LD78 gene and mapping of all three LD78 gene loci to human chromosome 17. *DNA Sequence*, **3**, 203-212.
HUSSUSSIAN CJ. STRUEWING JP. GOLDSTEIN AM. HIGGINS PAT. ALLY DS. SHEAHAN MD. CLARK Jr WH. TUCKER MA AND DRACOPOLI NC. (1994). Germline *p16* mutations in familial melanoma. *Nature Genet.*, **8**, 15-21.
JEN J. HARPER JW. BIGNER SH. BIGNER DD. PAPADOPOULOS N. MARKOWITZ S. WILLSON JKV. KINZLER KW AND VOGELSTEIN B. (1994). Deletion of *p16* and *p15* genes in brain tumors. *Cancer Res.*, **54**, 6353-6358.
JUNIEN C AND HENRY I. (1994). Genetics of Wilms' tumour: a blend of aberrant development and genomic imprinting. *Kidney Int.*, **46**, 1264-1279.
KAMB A. LIU Q. HARSHMAN K. TAVTIGIAN S. CORDON-CARDO C AND SKOLNICK MH. (1994*a*). Rates of *p16* (MTS1) mutations in primary tumors with 9p loss (reply). *Science*, **265**, 416-417.
KAMB A. GRUIS NA. WEAVER-FELDHAUS J. LIU Q. HARSHMAN K. TAVTIGIAN SV. STOCKERT E. DAY RS. JOHNSON BE AND SKOLNICK MH. (1994*b*). A cell cycle regulator potentially involved in genesis of many tumor types. *Science*, **264**, 436-440.
KEEN AJ AND KNOWLES MA. (1994). Definition of two regions of deletion on chromosome 9 in carcinoma of the bladder. *Oncogene*, **9**, 2083-2088.
MARX J. (1994*a*). How cells cycle toward cancer. *Science*, **263**, 319-321.
MARX J. (1994*b*). A challenge to *p16* gene as a major tumor suppressor. *Science*, **264**, 1846.
MASTERS JRW. HEPBURN PJ. WALKER L. HIGHMAN WJ. TREJDOSIEWICZ LK. POVEY S. PARKAR M. HILL BT. RIDDLE PR AND FRANKS LM. (1986). Tissue culture model of transitional cell carcinoma: characterization of twenty-two human urothelial cell lines. *Cancer Res.*, **46**, 3630-3636.
MASTERS JRW. BEDFORD P. KEARNEY A. POVEY S AND FRANKS LM. (1988). Bladder cancer cell line cross-contamination: identification using a locus-specific minisatellite probe. *Br. J. Cancer*, **57**, 284-286.
MIYAO N. TSAI YC. LERNER SP. OLUMI AF. SPRUCK III CH. GONZALEZ-ZULUETA M. NICHOLS PW. SKINNER DG AND JONES PA. (1993). Role of chromosome 9 in human bladder cancer. *Cancer Res.*, **53**, 4066-4070.
NIELL HB AND SOLOWAY MS. (1983). Use of the tumour colony assay in the evaluation of patients with bladder cancer. *Br. J. Urol.*, **55**, 271-274.
NOBORI T. MIURA K. WU DJ. LOIS A. TAKABAYASHI K AND CARSON DA. (1994). Deletions of the cyclin-dependent kinase-4 inhibitor gene in multiple human cancers. *Nature*, **368**, 753-756.
NODA A. NING Y. VENABLE SF *et al.* (1994). Cloning of senescent cell-derived inhibitors of DNA synthesis using an expression screen. *Exp. Cell Res.*, **211**, 90-98.
POLYAK K. LEE M-H. ERDJUMENT-BROMAGE H. KOFF A. ROBERTS JM. TEMPST P AND MASSAGUE J. (1994*a*). Cloning of *p27*$^{kip1}$, a cyclin-dependent kinase inhibitor and a potential mediator of extracellular antimitogenic signals. *Cell*, **78**, 59-66.
POLYAK K. KATO J-Y. SOLOMON MJ. SHERR CJ. MASSAGUE J. ROBERTS JM AND KOFF A. (1994*b*). p27$^{kip1}$, a cyclin-Cdk inhibitor, links transforming growth factor-$\beta$ and contact inhibition to cell cycle arrest. *Genes Dev.*, **8**, 9-22.
O'TOOLE CM. POVEY S. HEPBURN P AND FRANKS LM. (1983). Identity of some human bladder cancer cell lines. *Nature*, **301**, 429-430.
RUPPERT JM. TOKINO K AND SIDRANSKY D. (1993). Evidence for two bladder cancer suppressor loci on human chromosome 9. *Cancer Res.*, **53**, 5093-5095.
SERRANO M. HANNON GJ AND BEACH D. (1993). A new regulatory motif in cell-cycle control causing specific inhibition of cyclin D/CDK4. *Nature*, **366**, 704-707.
SIDRANSKY D. VON ESCHENBACH A. TSAI YC. JONES P. SUMMERHAYES I. MARSHALL F. PAUL M. GREEN P. HAMILTON SR. FROST P AND VOGELSTEIN B. (1991). Identification of p53 mutations in bladder cancers and urine samples. *Science*, **252**, 706-709.
SOUTHGATE J. HUTTON KAR. THOMAS DFM AND TREJDOSIEWICZ LK. (1994). Normal human urothelial cells *in vitro*: proliferation and induction of stratification. *Lab. Invest.*, **71**, 583-594.
SPRUCK III CH. GONZALEZ-ZULUETA M. SHIBATA A. SIMONEAU AR. LIN M-F. GONZALES F. TSAL YC AND JONES PA. (1994). *p16* gene in uncultured tumours. *Nature*, **370**, 183-184.
STADLER WM. SHERMAN J. BOHLANDER SK. ROULSTON D. DREYLING M. RUKSTALIS D AND OLOPADE OI. (1994). Homozygous deletions within chromosome bands 9p21-22 in bladder cancer. *Cancer Res.*, **54**, 2060-2063.
TOYOSHIMA H AND HUNTER T. (1994). *p27*, a novel inhibitor of $G_1$ cyclin-Cdk protein kinase activity, is related to *p21*. *Cell*, **78**, 67-74.
TREJDOSIEWICZ LK. SOUTHGATE J. DONALD JA. MASTERS JRW. HEPBURN PJ AND HODGES GM. (1985). Monoclonal antibodies to human urothelial cell lines and hybrids: production and characterization. *J. Urol.*, **133**, 533-538.
WAINWRIGHT B. (1994). Familial melanoma and *p16*. A hung jury. *Nature Genet.*, **8**, 3-5.
XIONG Y. HANNON GJ. ZHANG H. CASSO D. KOBAYASHI R AND BEACH D. (1993*a*). *p21* is a universal inhibitor of cyclin kinases. *Nature*, **366**, 701-704.
XIONG Y. ZHANG H AND BEACH D. (1993*b*). Subunit rearrangement of the cyclin-dependent kinases is associated with cellular transformation. *Genes Dev.*, **7**, 1572-1583.
YEAGER T. STADLER W. BELAIR C. PUTHENVEETTIL J. OLOPADE O AND REZNIKOFF C. (1995). Increased p16 levels correlate with pRb alterations in human urothelial cells. *Cancer Res.*, **55**, 493-497.


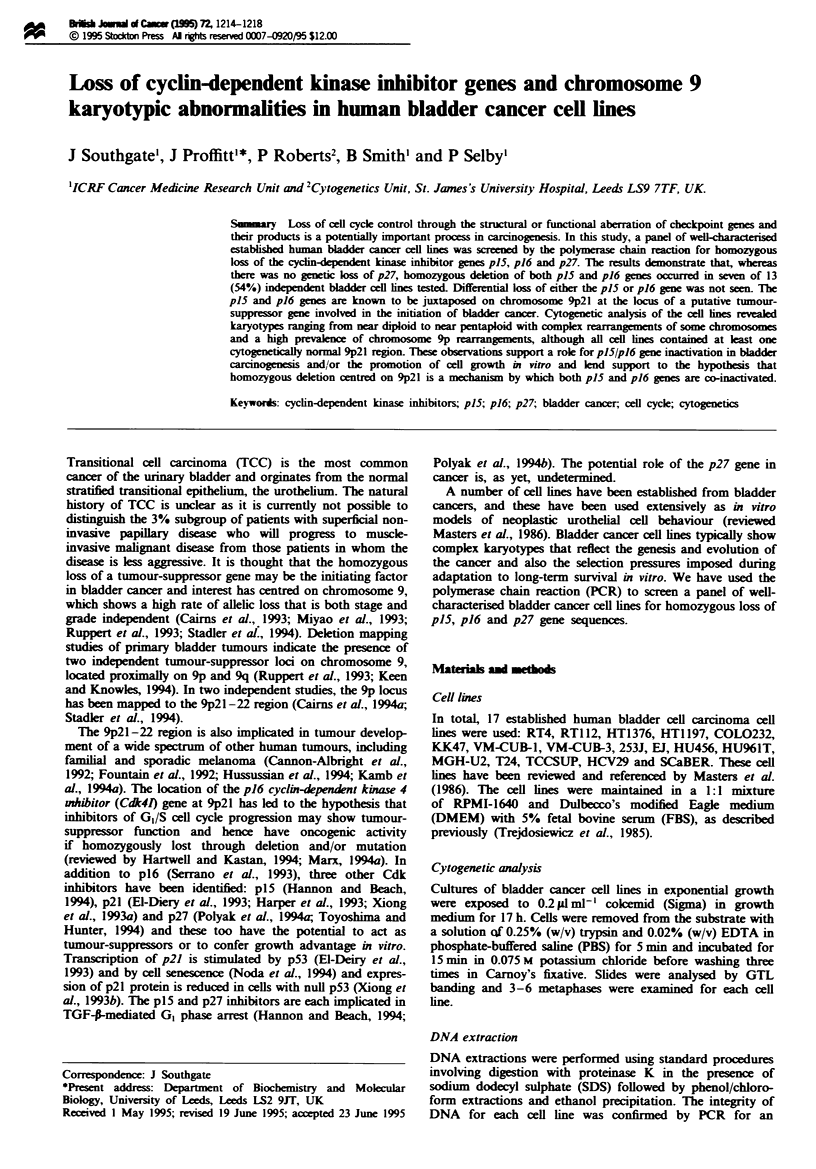

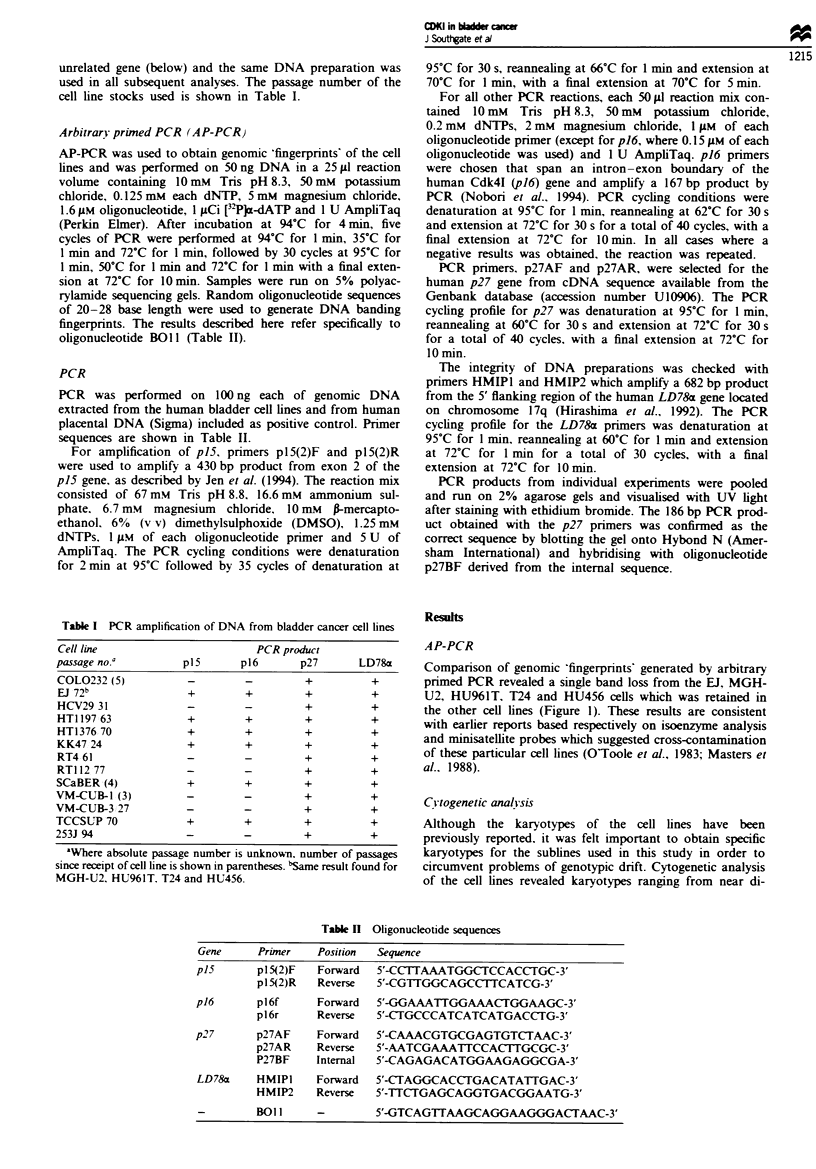

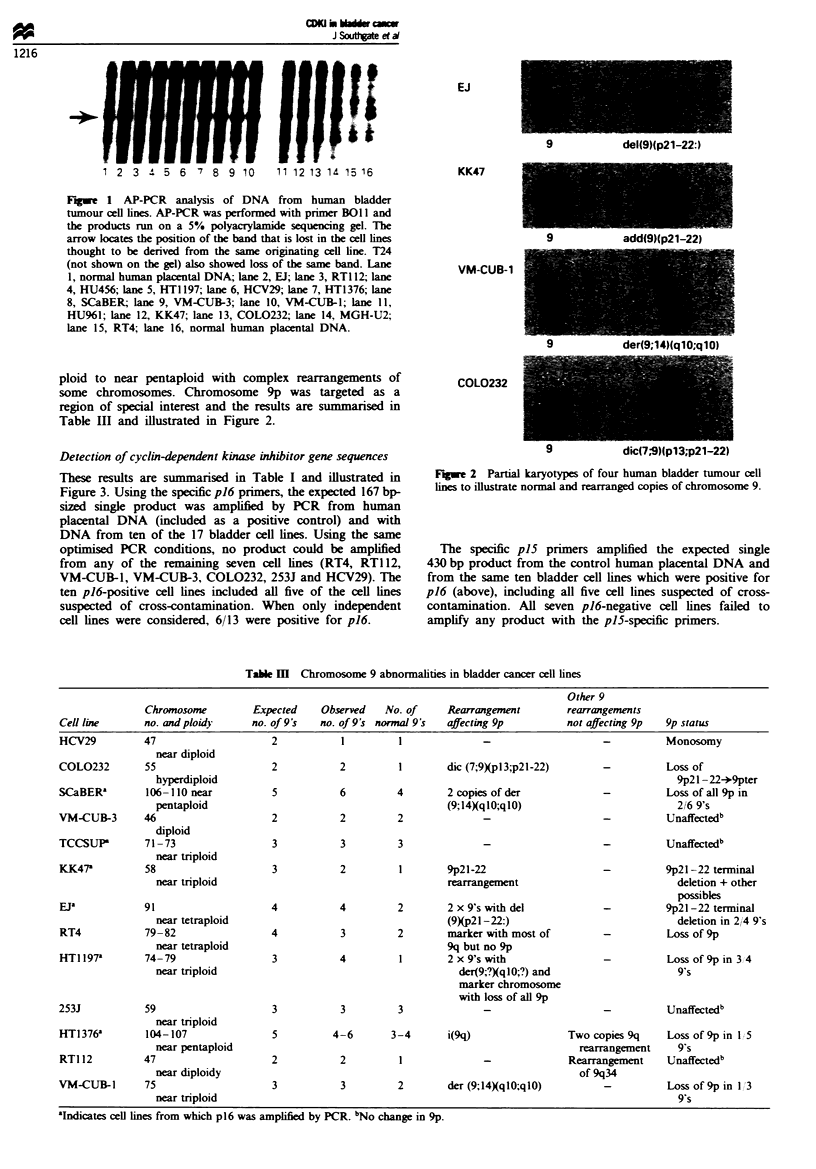

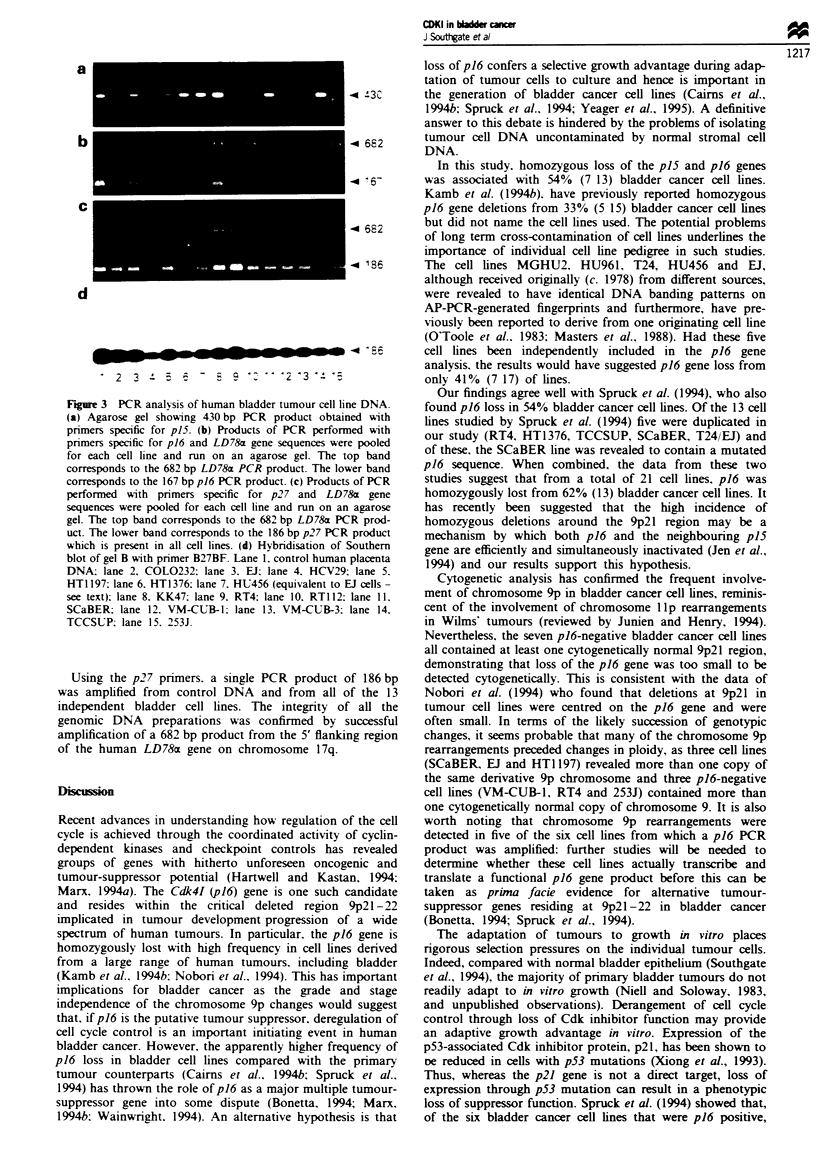

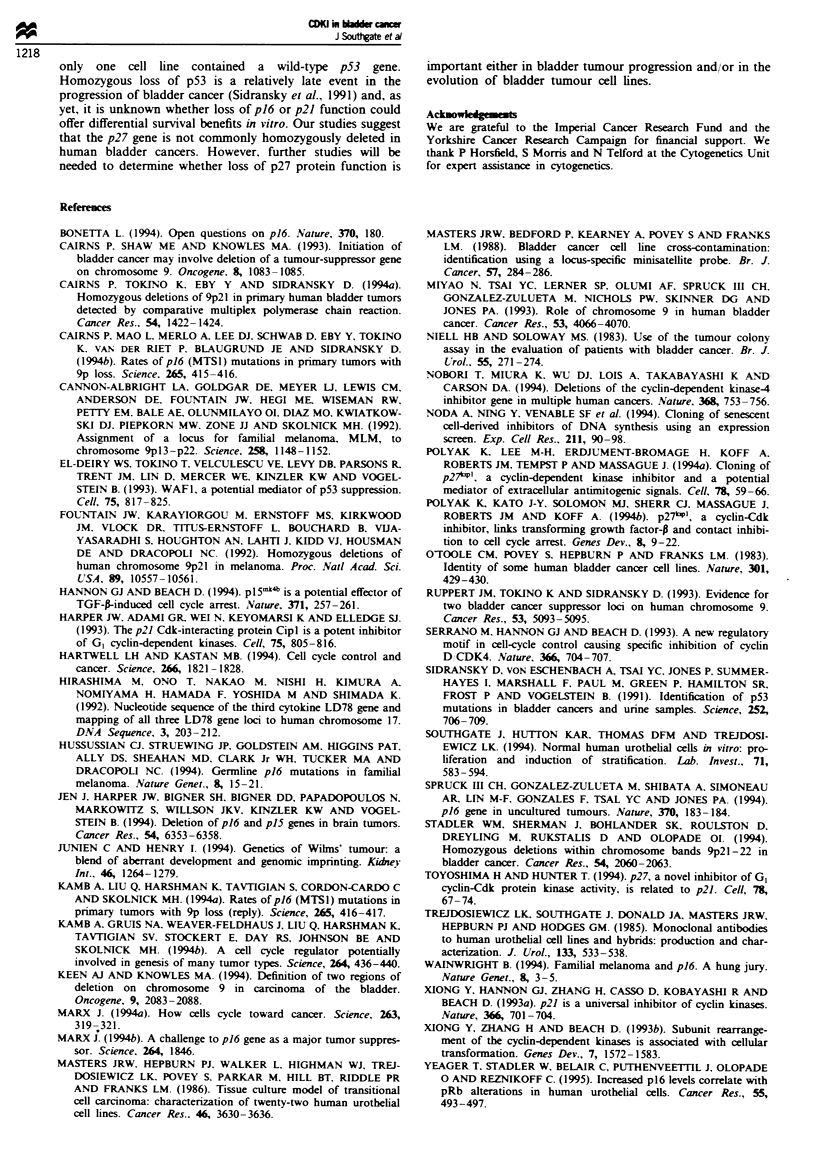


## References

[OCR_00597] Bonetta L. (1994). Tumor-suppressor genes. Open questions on p16.. Nature.

[OCR_00610] Cairns P., Mao L., Merlo A., Lee D. J., Schwab D., Eby Y., Tokino K., van der Riet P., Blaugrund J. E., Sidransky D. (1994). Rates of p16 (MTS1) mutations in primary tumors with 9p loss.. Science.

[OCR_00601] Cairns P., Shaw M. E., Knowles M. A. (1993). Initiation of bladder cancer may involve deletion of a tumour-suppressor gene on chromosome 9.. Oncogene.

[OCR_00606] Cairns P., Tokino K., Eby Y., Sidransky D. (1994). Homozygous deletions of 9p21 in primary human bladder tumors detected by comparative multiplex polymerase chain reaction.. Cancer Res.

[OCR_00616] Cannon-Albright L. A., Goldgar D. E., Meyer L. J., Lewis C. M., Anderson D. E., Fountain J. W., Hegi M. E., Wiseman R. W., Petty E. M., Bale A. E. (1992). Assignment of a locus for familial melanoma, MLM, to chromosome 9p13-p22.. Science.

[OCR_00633] Fountain J. W., Karayiorgou M., Ernstoff M. S., Kirkwood J. M., Vlock D. R., Titus-Ernstoff L., Bouchard B., Vijayasaradhi S., Houghton A. N., Lahti J. (1992). Homozygous deletions within human chromosome band 9p21 in melanoma.. Proc Natl Acad Sci U S A.

[OCR_00640] Hannon G. J., Beach D. (1994). p15INK4B is a potential effector of TGF-beta-induced cell cycle arrest.. Nature.

[OCR_00642] Harper J. W., Adami G. R., Wei N., Keyomarsi K., Elledge S. J. (1993). The p21 Cdk-interacting protein Cip1 is a potent inhibitor of G1 cyclin-dependent kinases.. Cell.

[OCR_00649] Hartwell L. H., Kastan M. B. (1994). Cell cycle control and cancer.. Science.

[OCR_00654] Hirashima M., Ono T., Nakao M., Nishi H., Kimura A., Nomiyama H., Hamada F., Yoshida M. C., Shimada K. (1992). Nucleotide sequence of the third cytokine LD78 gene and mapping of all three LD78 gene loci to human chromosome 17.. DNA Seq.

[OCR_00661] Hussussian C. J., Struewing J. P., Goldstein A. M., Higgins P. A., Ally D. S., Sheahan M. D., Clark W. H., Tucker M. A., Dracopoli N. C. (1994). Germline p16 mutations in familial melanoma.. Nat Genet.

[OCR_00664] Jen J., Harper J. W., Bigner S. H., Bigner D. D., Papadopoulos N., Markowitz S., Willson J. K., Kinzler K. W., Vogelstein B. (1994). Deletion of p16 and p15 genes in brain tumors.. Cancer Res.

[OCR_00672] Junien C., Henry I. (1994). Genetics of Wilms' tumor: a blend of aberrant development and genomic imprinting.. Kidney Int.

[OCR_00680] Kamb A., Gruis N. A., Weaver-Feldhaus J., Liu Q., Harshman K., Tavtigian S. V., Stockert E., Day R. S., Johnson B. E., Skolnick M. H. (1994). A cell cycle regulator potentially involved in genesis of many tumor types.. Science.

[OCR_00678] Kamb A., Liu Q., Harshman K., Tavtigian S., Cordon-Cardo C., Skolnick M. H. (1994). Response.. Science.

[OCR_00687] Keen A. J., Knowles M. A. (1994). Definition of two regions of deletion on chromosome 9 in carcinoma of the bladder.. Oncogene.

[OCR_00696] Marx J. (1994). A challenge to p16 gene as a major tumor suppressor.. Science.

[OCR_00692] Marx J. (1994). How cells cycle toward cancer.. Science.

[OCR_00708] Masters J. R., Bedford P., Kearney A., Povey S., Franks L. M. (1988). Bladder cancer cell line cross-contamination: identification using a locus-specific minisatellite probe.. Br J Cancer.

[OCR_00701] Masters J. R., Hepburn P. J., Walker L., Highman W. J., Trejdosiewicz L. K., Povey S., Parkar M., Hill B. T., Riddle P. R., Franks L. M. (1986). Tissue culture model of transitional cell carcinoma: characterization of twenty-two human urothelial cell lines.. Cancer Res.

[OCR_00715] Miyao N., Tsai Y. C., Lerner S. P., Olumi A. F., Spruck C. H., Gonzalez-Zulueta M., Nichols P. W., Skinner D. G., Jones P. A. (1993). Role of chromosome 9 in human bladder cancer.. Cancer Res.

[OCR_00717] Niell H. B., Soloway M. S. (1983). Use of the tumour colony assay in the evaluation of patients with bladder cancer.. Br J Urol.

[OCR_00722] Nobori T., Miura K., Wu D. J., Lois A., Takabayashi K., Carson D. A. (1994). Deletions of the cyclin-dependent kinase-4 inhibitor gene in multiple human cancers.. Nature.

[OCR_00726] Noda A., Ning Y., Venable S. F., Pereira-Smith O. M., Smith J. R. (1994). Cloning of senescent cell-derived inhibitors of DNA synthesis using an expression screen.. Exp Cell Res.

[OCR_00744] O'Toole C. M., Povey S., Hepburn P., Franks L. M. (1983). Identity of some human bladder cancer cell lines.. Nature.

[OCR_00736] Polyak K., Kato J. Y., Solomon M. J., Sherr C. J., Massague J., Roberts J. M., Koff A. (1994). p27Kip1, a cyclin-Cdk inhibitor, links transforming growth factor-beta and contact inhibition to cell cycle arrest.. Genes Dev.

[OCR_00731] Polyak K., Lee M. H., Erdjument-Bromage H., Koff A., Roberts J. M., Tempst P., Massagué J. (1994). Cloning of p27Kip1, a cyclin-dependent kinase inhibitor and a potential mediator of extracellular antimitogenic signals.. Cell.

[OCR_00749] Ruppert J. M., Tokino K., Sidransky D. (1993). Evidence for two bladder cancer suppressor loci on human chromosome 9.. Cancer Res.

[OCR_00754] Serrano M., Hannon G. J., Beach D. (1993). A new regulatory motif in cell-cycle control causing specific inhibition of cyclin D/CDK4.. Nature.

[OCR_00759] Sidransky D., Von Eschenbach A., Tsai Y. C., Jones P., Summerhayes I., Marshall F., Paul M., Green P., Hamilton S. R., Frost P. (1991). Identification of p53 gene mutations in bladder cancers and urine samples.. Science.

[OCR_00767] Southgate J., Hutton K. A., Thomas D. F., Trejdosiewicz L. K. (1994). Normal human urothelial cells in vitro: proliferation and induction of stratification.. Lab Invest.

[OCR_00770] Spruck C. H., Gonzalez-Zulueta M., Shibata A., Simoneau A. R., Lin M. F., Gonzales F., Tsai Y. C., Jones P. A. (1994). p16 gene in uncultured tumours.. Nature.

[OCR_00778] Stadler W. M., Sherman J., Bohlander S. K., Roulston D., Dreyling M., Rukstalis D., Olopade O. I. (1994). Homozygous deletions within chromosomal bands 9p21-22 in bladder cancer.. Cancer Res.

[OCR_00783] Toyoshima H., Hunter T. (1994). p27, a novel inhibitor of G1 cyclin-Cdk protein kinase activity, is related to p21.. Cell.

[OCR_00786] Trejdosiewicz L. K., Southgate J., Donald J. A., Masters J. R., Hepburn P. J., Hodges G. M. (1985). Monoclonal antibodies to human urothelial cell lines and hybrids: production and characterization.. J Urol.

[OCR_00798] Xiong Y., Hannon G. J., Zhang H., Casso D., Kobayashi R., Beach D. (1993). p21 is a universal inhibitor of cyclin kinases.. Nature.

[OCR_00801] Xiong Y., Zhang H., Beach D. (1993). Subunit rearrangement of the cyclin-dependent kinases is associated with cellular transformation.. Genes Dev.

[OCR_00806] Yeager T., Stadler W., Belair C., Puthenveettil J., Olopade O., Reznikoff C. (1995). Increased p16 levels correlate with pRb alterations in human urothelial cells.. Cancer Res.

[OCR_00624] el-Deiry W. S., Tokino T., Velculescu V. E., Levy D. B., Parsons R., Trent J. M., Lin D., Mercer W. E., Kinzler K. W., Vogelstein B. (1993). WAF1, a potential mediator of p53 tumor suppression.. Cell.

